# Formation of the sacrum requires down-regulation of sonic hedgehog signaling in the sacral intervertebral discs

**DOI:** 10.1242/bio.035592

**Published:** 2018-05-21

**Authors:** Raffaella Bonavita, Kathleen Vincent, Robert Pinelli, Chitra Lekha Dahia

**Affiliations:** 1 Orthopaedic Soft Tissue Research Program, Hospital for Special Surgery, New York, NY 10021, USA; 2 Department of Cell and Developmental Biology, Weill Cornell Medical College, New York, NY 10065, USA

**Keywords:** SHH, Brachyury, SmoM2, Regeneration, Sacrum disc, Vascularization

## Abstract

In humans, the sacrum forms an important component of the pelvic arch, and it transfers the weight of the body to the lower limbs. The sacrum is formed by collapse of the intervertebral discs (IVDs) between the five sacral vertebrae during childhood, and their fusion to form a single bone. We show that collapse of the sacral discs in the mouse is associated with the down-regulation of sonic hedgehog (SHH) signaling in the nucleus pulposus (NP) of the disc, and many aspects of this phenotype can be reversed by experimental postnatal activation of hedgehog (HH) signaling. We have previously shown that SHH signaling is essential for the normal postnatal growth and differentiation of intervertebral discs elsewhere in the spine, and that loss of SHH signaling leads to pathological disc degeneration, a very common disorder of aging. Thus, loss of SHH is pathological in one region of the spine but part of normal development in another.

## Abstract (precis)

**Summary:** Loss of SHH signaling is associated with the collapse of the sacral discs and formation of the sacrum. Conditional reactivation of SHH re-awakens the sacral disc, suggesting its potential for disc regeneration.

## INTRODUCTION

Intervertebral discs (IVDs) are cartilaginous structures present between each vertebral body. They have three main components; a central core of reticular nucleus pulposus (NP) cells derived from the embryonic notochord (Carlier, 1890; Choi et al., 2008; Peacock, 1951, 1952; Walmsley, 1953), surrounded by orthogonal layers of the fibrocartilaginous annulus fibrosus (AF) derived from the syndetome (Murchison et al., 2007; Sugimoto et al., 2013) region of the somite, and cartilaginous endplates (CEP or EP) which separate the NPs from adjacent vertebral bodies. Once formed, the IVDs continue to be present between each vertebra during childhood, but become fused between the sacral vertebrae in humans by the time of adolescence to form the sacrum (Gray, 2008).

We have previously shown that sonic hedgehog (SHH) secreted by the NP cells during postnatal stages is crucial for the maintenance of postnatal mouse lumbar discs, including cell proliferation, maintenance of NP markers Brachyury (BRA) (Dahia et al., 2009b) and Cytokeratin 19 (CK19), and extracellular matrix (ECM) production (Dahia et al., 2012). SHH signaling is reduced with age in the lumbar vertebrae, causing the age-related collapse of the NP cells and loss of ECM in the entire disc (Dahia et al., 2009a; Winkler et al., 2014).

Given that the NP collapses between the sacral vertebrae, we tested the hypothesis that SHH loss is physiological in the formation of the sacrum. First, we show that SHH expression is down-regulated in postnatal sacral discs. Second, using a conditional mouse model to activate hedgehog (HH) signaling in the NP cells, we show that reactivation of HH signaling results in reactivation of the dormant NP cells and re-activation of the sacral discs.

## RESULTS AND DISCUSSION

### Formation of the mouse sacrum

**Fig. 1.**
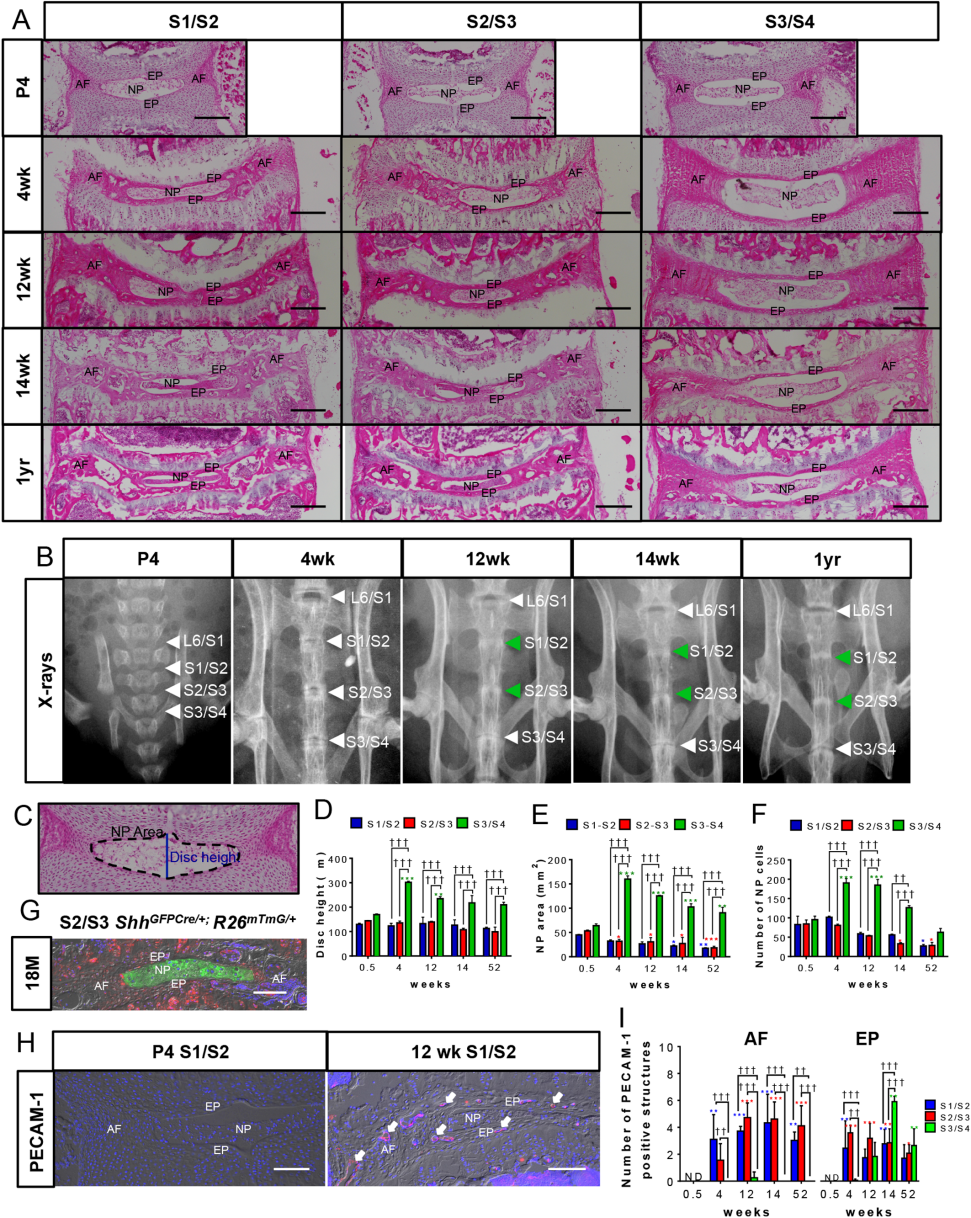
**Formation of mouse sacrum.** (A) Images are arranged in a tabular form with H and E-stained mid-coronal sections of the three levels of sacral discs (columns) from male FVB mice at P4, 4 weeks, 12 weeks, 14 weeks, and 1 year of age (rows). (B) X-ray images of the sacral spine at ages corresponding to A. Collapse of S1-S3 discs suggested by the loss of dark area between these discs at 12 weeks onwards is indicated by green arrows. (C) The schema for morphometric analysis. (D-F) Graphs representing quantification of the morphometric parameters; measurement for disc height (D); NP area (E); number of NP cells quantified in this study (F). (G) The S2/S3 disc from 18-month old *Shh^GFPCre/+^; R26^mTmG/+^* line. Green cells are derived from *Shh-* expressing population. (H) S1/S2 discs from P4 and 12 weeks stained for PECAM-1. (I) Number of PECAM-1+ vascular structures in AF and EP with aging. Mean±s.e.m., *N*=3 each, for each time point. Significance was calculated using two-way ANOVA. ††*P*<0.01, †††*P*<0.001 compares data under bars, *P<0.05, **P<0.01, ***P<0.001 compares data under asterisk to its P4 value. Scale bars: 200 μm in A, 100 μm in G and H. Images in B are presented not to scale. Nuclei are counter-stained with DAPI in G and H and images are captured using DIC filter.

Most strains of mice have seven cervical, thirteen thoracic, five to six lumbar, and four sacral vertebrae (McLaren and Michie, 1958; Wellik, 2007). To determine the timeline of sacrum formation in mice, the sacral discs (S1/S2, S2/S3, S3/S4) of male FVB mice at a range of ages from neonatal (postnatal day 4, P4), rapid growth phase (4 weeks), end of longitudinal growth period (12 to 14 weeks) to middle-age (1 year old), were compared by X-ray (Fig. 1B) and histomorphometric analysis of Hematoxylin and Eosin (H and E)-stained mid-coronal sections (Fig. 1A,C-F). All sacral discs had normal histology at P4 with reticular NP cells and well-organized layers of AF (Fig. 1A). At 4 weeks of age, all sacral discs continued to have defined reticular NP, whereas the AF was disorganized in the two most cranial sacral discs (S1/S2 and S2/S3). By 12 to 14 weeks of age, the NP cells had collapsed in the two cranial discs and the disc height and NP spaces were dramatically reduced compared with S3/S4 (compare S1/S2 with S3/S4 in Fig. 1A,D,E). The AF of these two cranial discs appeared more disorganized, had no detectable layers and contained structures that appeared like vascular bodies, which are known to appear during age-related disc degeneration (Kauppila, 1995; Nerlich et al., 2007). The presence of vascular bodies was confirmed and quantified for each disc by immunostaining for the endothelial cell marker CD31 (PECAM-1, Fig. 1H,I). Immunofluorescence staining for PECAM-1 confirmed the presence of vascular bodies in the AF of S1/S2 and S2/S3 as early as 4 weeks of age (Fig. 1I). No PECAM-1+ structures were observed in the NP.

In contrast to the two cranial sacral discs, the S3/S4 disc maintained a normal histology and did not fuse at any of the stages studied and maintained all components of the disc: central NP, layered AF, and EP (Fig. 1A). The only change observed over time was the appearance of PECAM-1+ vascular bodies in the EP starting at 12 weeks of age (Fig. 1I). X-rays showed a progressive decline in the spaces (green arrowheads in Fig. 1B) between the S1/S2, and S2/S3 discs 4 weeks onwards, while the S3/S4 disc space was maintained at all stages that were analyzed (white arrowheads in Fig. 1B). Using a two-way between group ANOVA for histomorphometric analysis, a significant interaction revealed that disc height (Fig. 1D), NP area (Fig. 1E) and the number of NP cells (Fig. 1F) was increased significantly in S3/S4 at all age points compared with P4 and this increase was significantly greater than in S1/S2 and S2/S3 levels. Although the number of NP cells was similar in all the three sacral discs at P4, an increase in number of NP cells from P4 to 4 weeks of age was observed only in the S3/S4 disc. This increase in cell number may be associated with more cells proliferating, as indicated by the Ki67+ immunostained NP cells (Fig. S2A), than dying, as indicated by TUNEL assay (Fig. S2B). Indeed, while at P4, all sacral levels had more Ki67+ than TUNEL+ cells, at 4 weeks only S3/S4 continued to have more proliferation than cell death (Fig. S2C). By 1 year of age, there were very few NP cells in the S1/S2 and S2/S3 discs, and they were all round and clumped together (Fig. 1A; Fig. S1). To confirm that the round and clumped cells were NP cells, we analyzed the mid-coronal sections of S2/S3 discs from 18-month-old *Shh^GFPcre/+^*; *R26^mTmG/+^* mice generated by crossing *Shh^GFPcre/+^* (Harfe et al., 2004); with *R26^tm4(ACTB-tdTomato,-EGFP)Luo^* (Muzumdar et al., 2007) lines, that showed the center of the disc green, confirming its origin from the earlier NP cells (Fig. 1G). Using an epi-fluorescence microscope, we did not detect natural fluorescence for GFP driven by *Shh* in 18-month-old *Shh^GFPcre/+^* discs (data not shown), indicating that the membrane-bound GFP detected in NP cells in Fig. 1G was due to *Cre*-mediated recombination of the *R26^mTmG/+^* transgene by *Shh^GFPcre/+^*.

### Loss of SHH and its targets is associated with the collapse of the sacral disc

**Fig. 2.**
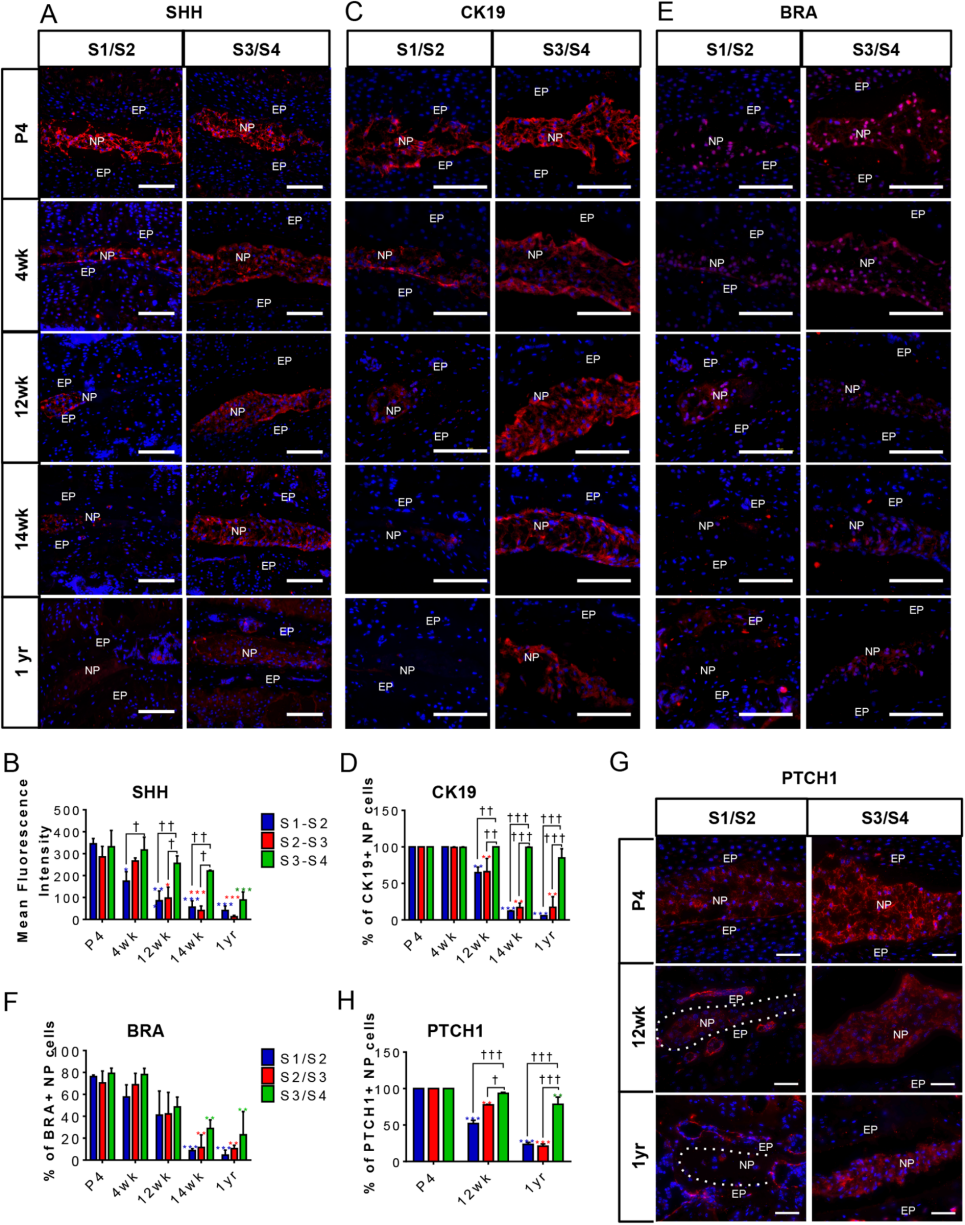
**Expression of SHH targets and notochordal markers in mouse sacral NP cells with aging.** (A-H) Immunofluorescence and quantification of notochordal/ NP markers: SHH (A,B); CK19 (C,D); BRA (E,F); PTCH1 (G,H) in the S1/S2, S2/S3 (quantification data only) and S3/S4 discs of male FVB mice at P4, 4 weeks, 12 weeks, 14 weeks, and 1 year of age. *N*=3 each. Scale bars: 100 μm. Nuclei are counter-stained with DAPI in all images. Mean±s.em. Two-way ANOVA. †*P*<0.05, ††*P*<0.01, †††*P*<0.001 compares data under bars, *P<0.05, **P<0.01, ***P<0.001 compares data under asterisk to its P4 value.

Previously, we have shown that SHH, produced by NP cells, is required for postnatal growth and differentiation of the disc, and that it decreases with age (Dahia et al., 2009a, 2012; Winkler et al., 2014). To determine whether fusion of the S1 to S3 sacral vertebrae is associated with a loss of SHH expression in the discs (or loss of signal response to SHH), immunostaining for SHH in S1/S2, S2/S3, and S3/S4 discs during growth and differentiation was carried out using male FVB mice. Fig. 2A,B shows a dramatic decrease in SHH expression from 4 weeks onwards in S1/S2; however, it decreased much more slowly in S3/S4, which maintained its normal structure longer. This result was also shown for the protein expression of the SHH target genes *Ck19*, *Bra* and *Ptch1*. The percentages of CK19-positive NP cells were reduced in the S1/S2 and S2/S3 (quantification data only) discs from 12 weeks onwards, compared with the S3/S4 disc where their expression was detectable until 1 year of age (Fig. 2C,D), corresponding to that of SHH. While BRA was reduced by 14 weeks onwards across all sacral levels (Fig. 2E,F), PTCH1 was specifically reduced in S1-S3 levels and not in S3/S4 at 12 weeks (Fig. 2G,H).

**Fig. 3.**
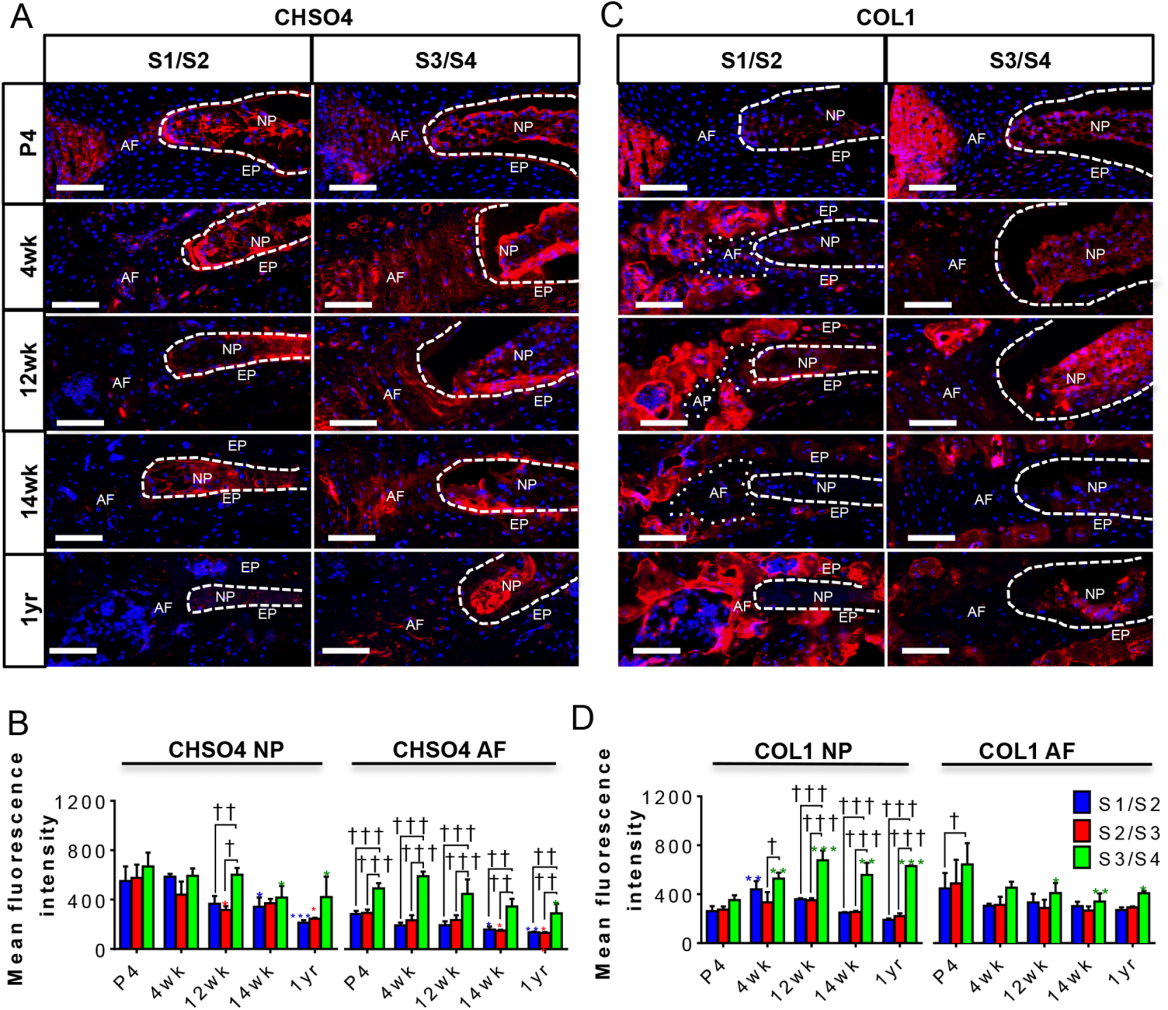
**Expression of SHH targets and ECM markers in sacral IVDs** **with aging.** (A-D) This figure is in a tabular form and shows the immunostaining and quantification of CHSO4 (A,B), and COL1 (C,D) in the S1/S2, S2/S3 (quantification data only) and S3/S4 discs of male FVB mice at P4, 4 weeks, 12 weeks, 14 weeks, and 1 year of age. *N*=3 each. Scale bars: 100 μm. Nuclei are counter-stained with DAPI in all images. Mean±s.e.m. Two-way ANOVA. †*P*<0.05, ††*P*<0.01, †††*P*<0.001 compares data under bars *P<0.05, **P<0.01, ***P<0.001 compares data under asterisk to its P4 value.

The ECM plays an important role in the maintenance of the structure and function of the notochord (reviewed by Stemple, 2005) and the discs (reviewed by Sivan et al., 2014; Urban and Roberts, 2003). The NP is rich in proteoglycans and glycosaminoglycan (GAGs) (Antoniou et al., 1996), while the AF is relatively rich in collagens, which form its fibrils (reviewed by Urban and Roberts, 2003). In our previous studies, we showed that SHH regulates the expression and synthesis of ECM markers, both in the NP and AF, during postnatal development and that expression of these markers is reduced with age (Dahia et al., 2012; Winkler et al., 2014). We therefore analyzed the expression of the ECM markers Chondroitin sulfate (CHSO4) and Collagen 1 (COL1) by immunofluorescence in all sacral discs during postnatal growth and differentiation. At P4, the NPs in all three sacral discs had comparable levels of CHSO4 (Fig. 3A,B) and COL1 (Fig. 3C,D); however, by 12 weeks, both ECM markers were significantly greater in S3/S4 than S1/S2 and S2/S3 (quantification data only) and continued to be present at 1 year of age. Changes in ECM markers were observed not only in the NP cells, which express *Shh*, but also in the surrounding AF of the sacral discs with age, indicating that both cell types respond to SHH produced by the NP cells directly or indirectly.

### Conditional activation of the HH pathway in NP cells can regenerate the sacral discs

**Fig. 4.**
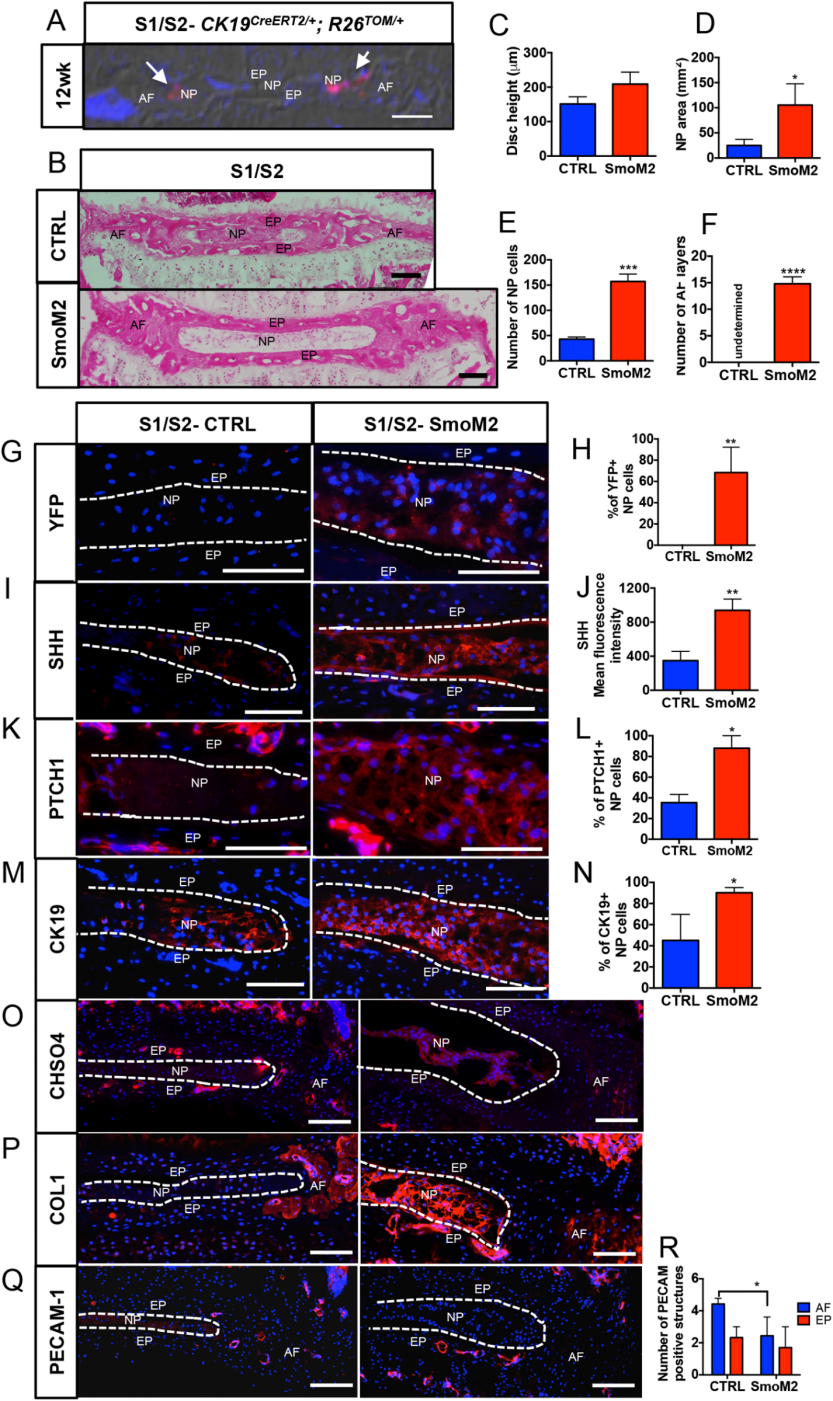
**Constitutive activation of SmoM2 in the NP cells re-activates the sacral disc.** (A) Coronal section of S1/S2 discs from *CK19^CreERT2/+^;* *R26^TOM/+^* gavaged at 11 weeks of age and analyzed 1 week later. Red cells are the *CK19^CreERT2/+^* expressing cells that have undergone recombination (white arrows). (B) H and E staining of the mid-coronal section from the control (CTRL- *R26^SmoM2-YFP/SmoM2-YFP^*) and SmoM2 (*CK19^CreERT2/+;^ R26^SmoM2-YFP/SmoM2-YFP^*) littermates. (C-F) Quantification of morphometric parameters disc height (C); area occupied by the NP cells (D); the number of NP cells (E); and the number of layers in the AF (F) in the sacral discs of the SmoM2 group compared to littermate controls. (G,H) Immunostaining and quantification of YFP+ NP cells in SmoM2 and control discs. (I-P) Data from immunostaining for SHH and its downstream targets and their quantification: SHH (I,J); PTCH1 (K,L); CK19 (M,N); CHSO4 (O); COL1 expression in S1/S2 discs from controls and SmoM2 mice (P). (Q,R) Immunostaining and quantification of PECAM-1 positive structures in the AF and EP of control and SmoM2 mouse discs. Mean±s.d. *N*=4 Controls; *N*=3 SmoM2. Unpaired two-tailed *t*-test. *P<0.05, **P<0.01, ***P<0.001, ****P<0.0001. Scale bars: 100 μm. Nuclei are counter-stained with DAPI in A, G, I, K, M, O, P and Q. A is captured using DIC filter.

Next, we tested whether the collapse of sacral discs can be reversed by re-activating SHH signaling at 12 weeks of age when the expression of SHH in the S1/S2 disc is already reduced and the NP cells have begun to lose their reticular phenotype, becoming rounder and clumped together. Since the *Ck19^CreERT2^* (Means et al., 2008) driver line was used to activate HH signaling, we determined how many cells expressed CreER and could undergo recombination by crossing it with a *Gt(ROSA)26Sortm^14(CAG-tdTomato)Hze^* (*R26^TOM/TOM^*) (Madisen et al., 2010) reporter to generate the *CK19^CreER/+^; R26^TOM/+^* line. Tamoxifen was administered to 11-week-old *CK19^CreER/+^; R26^TOM/+^* mice twice, 2 days apart, and the sacral spine was collected at 12 weeks of age. Mid-coronal sections were analyzed through the S1/S2 disc. Fig. 4A shows that only a small percentage of NP cells could be recombined using *CK19^CreERT2^* by 12 weeks of age in the S1/S2 discs (shown by white arrowheads). This observation is in line with data presented in Fig. 2C which show that not all NP cells in the S1/S2 discs expressed CK19 at 12 weeks of age. Next, we crossed *CK19^CreERT2/+^* with *R26^LSL-SmoM2-YFP/LSL-SmoM2-YFP^* (Jeong et al., 2004) to generate *CK19^CreERT2/+;^ R26^LSL-SmoM2-YFP/LSL-SmoM2-YFP^* (SmoM2) experimental and *R26^LSL-SmoM2-YFP/LSL-SmoM2-YFP^* (CTRL) control mice. Tamoxifen was administered to 12-week-old littermates twice, 2 days apart, to induce expression of the constitutively active form of the HH receptor SMO (*caSmo*), commonly known as SmoM2, in the sub-set of NP cells that express *CK19* (shown in Fig. 4A). The mice were euthanized, and the sacral spines collected 2 weeks after the last tamoxifen dose, when the mice were 14.5 weeks of age. Fig. 4B and Fig. S3 show a dramatic rescue of the S1/S2 disc based on histology in the SmoM2 experimental group compared to the littermate controls. All the NP cells had become reticular and evenly distributed, and the AF was more organized in the SmoM2 group. Histomorphometric analysis showed quantifiable differences that included improvement in disc height (Fig. 4C), NP area (*P*=0.0160, Fig. 4D), and an increase in the number of NP cells (*P*=0.0003) (Fig. 4E), and decreased cell death in the NP cells (Fig. S4) in the SmoM2 group compared to littermate controls. The AF of SmoM2 discs also had more defined layers (Fig. 4F). TUNEL assay showed a significant (*P*=0.0432) reduction in cell death of NP cells (Fig. 4A-C) following activation of HH signaling in the S1/S2 discs of SmoM2 mice compared to the controls. Interestingly, immunostaining for YFP (fused to SmoM2) showed that over 75% of NP cells were positive (Fig. 4G,H). Thus, since only a sub-set of NP cells normally expresses *CK19^CreERT2^* in the S1/S2 sacral disc at 12 weeks (Fig. 4A), these results suggest an expansion of the *SmoM2-*expressing NP cells. Furthermore, irrespective of being YFP+, all NP cells in the SmoM2 experimental discs were reticular. Hence, we analyzed the expression of SHH and found it to be higher in the SmoM2 discs (Fig. 4I,J), supporting the hypothesis that induction of SmoM2 expression stimulates SHH expression by NP cells; the sacral discs then respond to high levels of SHH irrespective of the whether these are the recombined cells expressing SmoM2 allele. This result also explains the rescue in the AF phenotype despite the expression of SmoM2 being driven exclusively in the NP cells. An increase in the downstream targets of SHH: PTCH1, CK19, CHSO4, and COL1 (Fig. 4K-P) was observed in the SmoM2 experimental discs. To determine whether vascularization of the AF in S1/S2 discs after 12 weeks of age (Fig. 1H,I) occurred as a result of decreased SHH levels, we quantified the number of PECAM-1+ vascular structures in the AF and EP of the experimental SmoM2 discs compared with controls. The results showed a significant decrease (*P*=0.0218) in the PECAM-1+ structures (Fig. 4Q,R) in the AF of the SmoM2 disc, although no change was observed in the EP. Vascularization of the AF has been described with aging and disc degeneration (Binch et al., 2014; Kauppila, 1995; Melrose et al., 2002; Nerlich et al., 2007), and has been related to loss of ECM, mostly proteoglycans (Johnson et al., 2005; Melrose et al., 2002).

## CONCLUSION

We have previously shown that SHH is important for the maintenance of the lumbar discs, and its loss is associated with the aging phenotype of the NP cells in these discs (Dahia et al., 2012; Winkler et al., 2014). Here, we show that there is a physiological loss of SHH in the sacrum when rest of the spine is still growing, that the loss of SHH is associated with collapse of the sacral discs and that activation of HH signaling can reverse many of the aspects of this phenotype. Moreover, a very interesting finding is that activation of HH signaling at 12 weeks in only a small proportion of NP cells (those that still express *CK19*), is sufficient to induce higher expression of SHH by the NP cells, which rescues the sacral disc. CK19 expression is specific to the NP cells in the IVDs (Fig. 4A and Dahia et al., 2012). It is also expressed in other cells in the body; the epithelial lining of the gut, for example. However, it is unlikely that the activation of SmoM2 in those cells will activate disc cells, which is the largest avascular structure in the body.

Our results are important because various approaches are being attempted to elicit regeneration of the intervertebral disc, including the use of stem cells, notochordal cells, or tissue engineering (reviewed in Kandel et al., 2008; Kregar Velikonja et al., 2014). However, our data show that activation of HH signaling, which can be achieved with a small molecule, even if only in a sub-set of dormant NP cells, can result in ‘re-activation’ of the entire disc by inducing *Shh* expression and secretion of the ligand. One of the limitations of the study is that the role of sex on the phenotype was not analyzed and requires further investigation.

The reasons for the normal collapse of only the most cranial sacral discs in the mouse are not understood. Something similar happens in humans, where all the sacral vertebrae fuse, but not the more caudal coccygeal vertebrae. The most plausible explanation is that the vertebrae with the greatest load fuse. In humans, the pelvis carries the weight of the whole body, whereas in mice it carries the weight of the caudal half only. Thus, the weight-bearing pelvic girdle might be made stronger in humans by the fusion of more vertebrae. How this difference in mechanical loading can cause the changes seen here is unknown. It will be interesting to know the extent to which SHH signaling in different discs along the craniocaudal axis is controlled by upstream patterning genes such as the Hox genes, or by mechanoreceptors.

## MATERIALS AND METHODS

### Mice

Wild-type male FVB, CK19^CreERT2/+^ (Means et al., 2008), R26^LSL-SmoM2-YFP/+^ (Jeong et al., 2004) Shh^GFPCre/+^ (Harfe et al., 2004), R26^tm4(ACTB-tdTomato,-EGFP)Luo^ (R26^mTmG^) (Muzumdar et al., 2007) and Gt(ROSA)26Sor^tm14(CAG-tdTomato)Hze^ (Madisen et al., 2010) mice of mixed backgrounds and of both sexes were used in this study. Tamoxifen (Sigma-Aldrich) was prepared in corn oil and administered by oral gavaging (6 mg/40 g body weight) twice, and 2 days apart, to adult mice. Mice were maintained, and all experiments were performed in accordance with Hospital for Special Surgery (HSS)- and Weill Cornell Medical College (WCMC)-approved protocols.

### Tissue preparation

Sacral vertebrae were dissected and fixed in 4% buffered Paraformaldehyde (PFA) for 2 h followed by decalcification using fresh 0.5 M Ethylenediaminetetraacetic acid (EDTA), pH 7.4, every day for 9 days at 4°C, before preparing molds and freezing in optimum cutting temperature (OCT) (VWR, Radnor, USA). Cryosections were prepared at 8 μm thickness in coronal plane using a cryostat (CM3050S; Leica, Wetzlar, Germany).

### Microscopy

Ni Eclipse (Nikon, Tokyo, Japan) inverted microscope was used for bright-field and fluorescence microscopy using NIS Elements software and 20× (dry), 40× (oil) and 60× (oil) objectives.

### Immunofluorescence staining

Immunostaining was performed as previously described (Dahia et al., 2012; Dahia et al., 2009a, b; Winkler et al., 2014). Briefly, following permeabilization using either buffered 0.5% Triton ×-100, or 10 mM Sodium Citrate for 20 min at room temperature, sections were blocked in blocking buffer for 1 hour and incubated with a specific primary antibody [GFP, 1:200, 11122, Life Technologies; CK19, 1:100, TROMA-II, Developmental Studies Hybridoma Bank (Iowa City, USA); SHH, 1:25, S4944, Sigma-Aldrich; PTCH1, 1:20, MAB41051, R&D Systems (Minneapolis, USA); Collagen1, 1:100, GTX41285, Genetex (Irvine, USA); CHSO4, 1:100, ab11570, Abcam; Ki67, 1:75, ab156611, Abcam; Brachyury, 1:10, ab156611, Abcam; CD31 (PECAM-1), 1:50, AF3628, R&D Systems] overnight at 4°C in a humidified chamber. The next day, the sections were washed and incubated with Alexa Fluor-conjugated secondary antibodies (Jackson-ImmunoResearch) diluted at 1:200 for 1 h and nuclei were counter-stained with DAPI (1:5000 in 1× PBS; D1306; Life Technologies) during the last wash. Slides were mounted using Prolong Gold mounting media (P36934; Life Technologies).

### Quantification and statistical analysis

Quantification of imaging data was carried out using NIS Elements software (Nikon). At least six H and E-stained mid-coronal serial sections were used for morphometric analysis and averages were taken. The changes in the disc height (H) and the area occupied by the NP cells in the sacral disc were measured as shown in Fig. 1C. DAPI+ nuclei were counted to determine the number of cells on three to six serial sections of immunostained slides. The percentages were determined by counting the number of DAPI+ cells that co-stained for the protein of interest (YFP+, CK19+, BRA+, PTCH1+, Ki67+ and TUNEL+). Imaging the sacral discs under 20× differential interference contrast (DIC) objective and counting each layer manually quantified changes in organization and AF layers. Mean fluorescence intensity was determined by selecting a region of interest corresponding specifically to NP and AF. PECAM-1+ structure in AF and EP were counted manually by combining fluorescence and 20× DIC objective.

Two to four mice were analyzed in each group. Data are presented as mean±s.e.m. (standard error of the mean). Statistical analysis was performed using Prism version 6.0f (GraphPad), either using an unpaired two-tailed *t-*test to compare two groups, or two-way, between-group ANOVA for comparisons between groups and over the period of time, with one factor the level in the sacrum (three levels: S1/S2, S2/S3, S3/S3) and the other factor age (five ages: P4, 4 weeks, 12 weeks, 14 weeks, 1 year). A significant main effect of either factor prompted post-hoc analyses of simple main effects within that specified factor. Simple main effects of age were assessed by comparing all ages within a disc level to that disc at P4. Dunnett's test was used to correct for multiple comparisons. Simple main effects of disc level were assessed by comparing between levels at a given age and Bonferroni's post-hoc test was used for multiple comparisons. A *P*-value of less than 0.05 was considered statistically significant. All significant post-hoc analyses are depicted in graphs with an asterisk (*) to indicate a significant change from P4 time points within the same sacral disc level, or a dagger (†) to indicate significant change between sacral disc levels at a particular age, unless otherwise specified. †*P*<0.05, ††*P*<0.01 and †††*P*<0.001 indicate significance between the data points under the line; *P<0.05, **P<0.01 and ***P<0.001 indicate significance between the data under the asterisk and the P4 value.

### X-ray imaging

Mice were sedated using 2-3% isoflurane at 1 l/min. X-ray images of the sacral spine were obtained from dorsal view at 5× resolution using the MX-20 Cabinet radiography system (Faxitron, Tucson, USA) by exposing each mouse for 10 s using the 26 kV high-resolution setting.

## Supplementary Material

Supplementary information
