# Supplementary material for: Formation of the sacrum requires down-regulation of sonic hedgehog signaling in the sacral intervertebral discs
Source: Biol Open. 2018 May 21;7(7):bio035592. doi: 10.1242/bio.035592 (PMC6078355; doi:10.1242/bio.035592)
Supplement: Supplementary information [file biolopen-7-035592-s1.pdf]

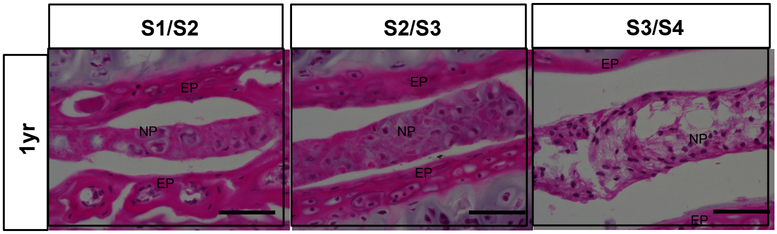

Figure S1. NP phenotype in 1yr old sacral discs. H and E stained coronal sections imaged at high magnification (600x) shows NP cells from sacral discs of one-year old mouse. All NP cells are seen clumped together and are round in S1/S2 and S2/S3 discs unlike the reticular NP cells seen in the S3/S4 discs that does not collapse with age. Images are representative of three biological sample analyzed. Scale bars= 50 µm.

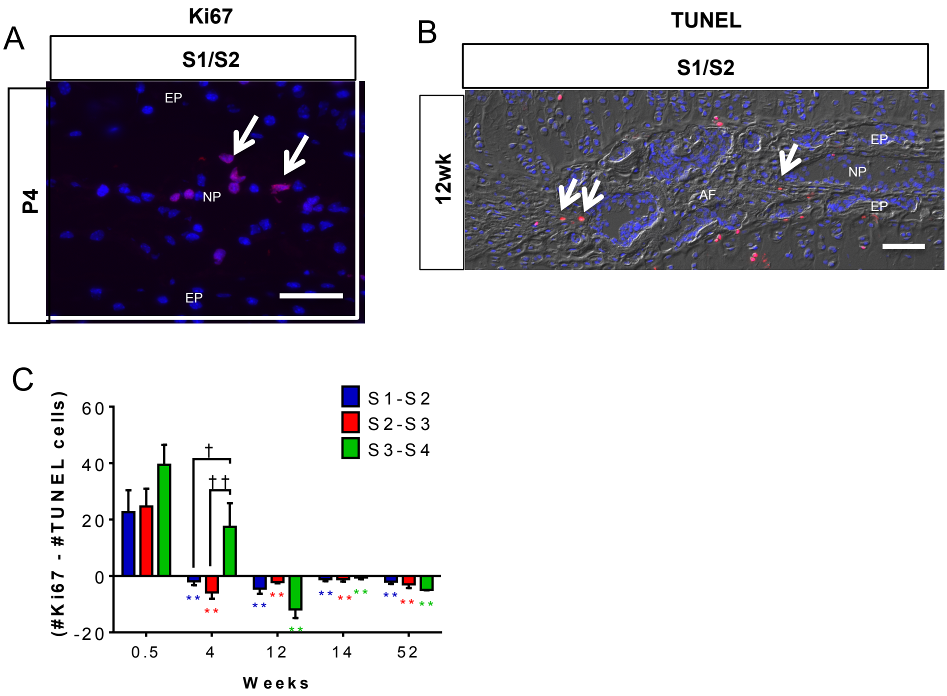

Cell growth and cell death

Ki67 immunostaining and quantification was performed as described in Material and Methods section.

TUNEL assay was carried out using *In Situ* Cell Death detection kit, TMR red (12156792910; Roche) as per manufacture's instructions. Briefly, sections were incubated with TUNEL reaction for 1h at 37°C in a humidified chamber. Next, the slides were washed, counterstained with DAPI, mounted with Pro-long Gold and imaged as described in the microscopy section.

Figure S2. Cell proliferation and cell death in mouse sacrum. (A) Representative image of Ki67 staining (white arrows) on P4 S1/S2 disc. (B) Representative image of TUNEL staining (white arrows) on 12 weeks S1/S2 discs. (C) Quantification of the "number of Ki67+ - number of TUNEL+ cells" in NP and AF for all sacral levels at different ages. Quantification of determining the of Ki67+ and TUNEL+ cells is described in Materials and Methods under "Quantification and Statistical Analysis" section. Mean±SEM. N=2-4 each. Two way, between group ANOVA, followed by post-hoc analysis for multiple comparison. †p<0.05, ††p<0.01, †††p<0.001 Bonferroni correction for pairwise comparisons between S1/S2, S2/S3 and S3/S4; *p<0.05, **p<0.01, ***p<0.001 Dunnett's correction for simple main effects of age vs. P4 and are in the same color as the respective sample. Scale bar in A= 50 µm, and B= 100 µm.

Biology Open • Supplementary information

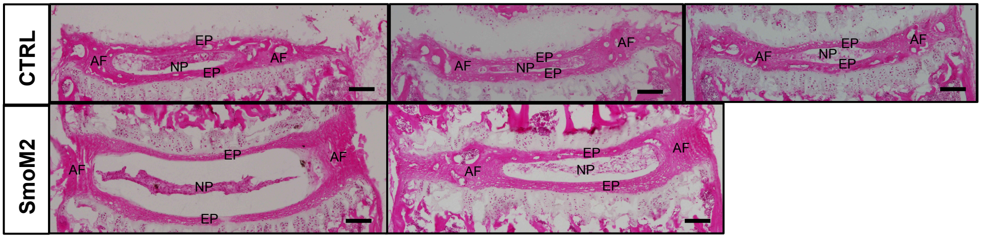

Figure S3. Rescue of S1/S2 disc following activation of SmoM2 in NP cells. H and E staining of the mid-coronal section of S1/S2 disc from *R26*$^{LSL\text{-}SmoM2\text{-}YFP/LSL\text{-}SmoM2\text{-}YFP}$ control (CTRL) and *CK19*$^{CreERT2/+}$; *R26*$^{LSL\text{-}SmoM2\text{-}YFP/LSL\text{-}SmoM2\text{-}YFP}$ (SmoM2) littermates. The images are representative of each biological replicate used in the study in addition to the ones shown in Fig. 4B. These H and E images show rescue of disc phenotype following activation of HH signaling in the NP cells of SmoM2 group, compared to the littermate controls.

Biology Open • Supplementary information

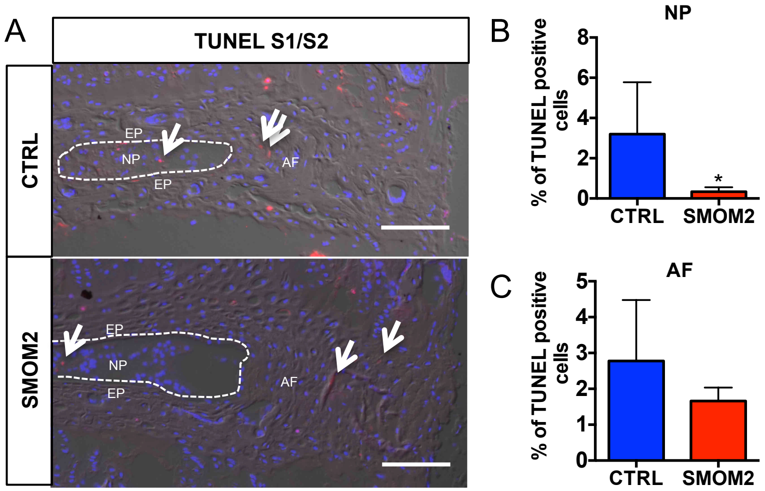

Figure S4. Activation of HH signaling by SmoM2 in the NP cells reduces cell death in sacral disc. (A) Representative image of TUNEL staining (white arrows) on S1/S2 discs from *R26*$^{LSL\text{-}SmoM2\text{-}YFP/LSL\text{-}SmoM2\text{-}YFP}$ control (CTRL) and *CK19*$^{CreERT2/+;}$ *R26*$^{LSL\text{-}SmoM2\text{-}YFP/LSL\text{-}SmoM2\text{-}YFP}$ (SmoM2) littermates. Quantification of the percentage of TUNEL+ cells in NP (B) and AF (C) is determined over the total number of cells in each region counted using DAPI. Mean± S.D. N=6 in each group by combining data from two independent experiments. Further details on quantification are provided in Materials and Methods under “Quantification and Statistical Analysis” section. Unpaired *t*-test. Scale bars= 100 μm.
